# The Keeping on Track Study: Exploring the Activity Levels and Utilization of Healthcare Services of Acute Coronary Syndrome (ACS) Patients in the First 30-Days after Discharge from Hospital

**DOI:** 10.3390/medsci7040061

**Published:** 2019-04-19

**Authors:** Robyn A. Clark, Jonathon Foote, Vincent L. Versace, Alex Brown, Mark Daniel, Neil T. Coffee, Tania S. Marin, Constance Kourbelis, Margaret Arstall, Anand Ganesan, Ralph Maddison, Janet Kelly, Tracey Barry, Wendy Keech, Stephen J. Nicholls, on behalf of the Health Translation SA Cardiac Rehabilitation Group

**Affiliations:** 1 College of Nursing and Health Sciences, Flinders University, Adelaide 5001, Australia; jon@footeconsulting.com.au (J.F.); tania.marin@flinders.edu.au (T.S.M.); constance.kourbelis@flinders.edu.au (C.K.); tracey.barry@flinders.edu.au (T.B.); 2 Deakin University, Warnambool, Victoria 3280, Australia; vincent.versace@deakin.edu.au (V.L.V.); ralph.maddison@deakin.edu.au (R.M.); 3 Wardliparingga Aboriginal Research Unit, South Australian Health and Medical Research Institute, Adelaide, South Australia 5001, Australia; alex.brown@sahmri.com (A.B.); Janet.Kelly@sahmri.com (J.K.); 4 Centre for Research and Action in Public Health, University of Canberra, Canberra, Australian Capital Territory 2001, Australia; Mark.Daniel@canberra.edu.au (M.D.); neil.coffee@canberra.edu.au (N.T.C.); 5 Department of Cardiology, Lyell McEwin Hospital, Elizabeth, South Australia 5112, Australia; Margaret.Arstall@sa.gov.au; 6 College of Medicine, Flinders University, Adelaide 5001, Australia; anand.ganesan@flinders.edu.au; 7 Health Translation SA, South Australian Health and Medical Research Institute, Adelaide, South Australia 5001, Australia; Wendy.keech@sahmri.com; 8 Monash University, Clayton, Victoria 3168, Australia; stephen.nicholls@monash.edu

**Keywords:** discharge education, physical activity, healthcare utilization, acute coronary syndrome, 30-days

## Abstract

The aim of this study was to investigate the impact of bedside discharge education on activity levels and healthcare utilization for patients with acute coronary syndrome (ACS) in the first 30 days post-discharge. Knowledge recall and objective activity and location data were collected by global positioning systems (GPS). Participants were asked to carry the tracking applications (apps) for 30–90 days. Eighteen participants were recruited (6 metropolitan 12 rural) 61% ST elevation myocardial infarction (STEMI), mean age 55 years, 83% male. Recall of discharge education included knowledge of diagnosis (recall = 100%), procedures (e.g., angiogram = 40%), and comorbidities (e.g., hypertension = 60%, diabetes = 100%). In the first 30 days post-discharge, median steps per day was 2506 (standard deviation (SD) ± 369) steps (one participant completed 10,000 steps), 62% visited a general practitioner (GP) 16% attended cardiac rehabilitation, 16% visited a cardiologist, 72% a pharmacist, 27% visited the emergency department for cardiac event, and 61% a pathology service (blood tests). Adherence to using the activity tracking apps was 87%. Managing Big Data from the GPS and physical activity tracking apps was a challenge with over 300,000 lines of raw data cleaned to 90,000 data points for analysis. This study was an example of the application of objective data from the real world to help understand post-ACS discharge patient activity. Rates of access to services in the first 30 days continue to be of concern.

## 1. Introduction

There has been considerable interest from hospitals and clinicians to better understand and improve modifiable factors associated with 30-day hospital readmissions [1,2,3,4,5]. However, there are limited data available describing relatively contemporary trends in 30-day prehospitalization among patients who survive hospitalization after an acute coronary syndrome (ACS) event [1,2,3]. A recent study from the United States of America has shown, of all cardiac readmissions within 30 days of hospitalization, the majority occurred within 15 days (67.6%, acute myocardial infarction and 61.0% heart failure). Neither readmission diagnoses nor timing substantively varied by age, sex, or race [6].

Hospitalized patients commonly become deconditioned during hospitalisation and, as a result, often develop impaired stamina, coordination, and strength, which place them at greater risk for accidents and falls [3]. These limitations may also diminish their ability to adhere to post-discharge instructions such as the capacity to resume basic activities or attend follow-up appointments. Although, most of the identified risk factors for 30-day readmissions are not readily modifiable (i.e., socioeconomics and comorbidities) understanding the gaps that exist in current post-discharge education practices will improve outcomes [1,2,3,4,5].

Currently, there is limited evidence for the effectiveness of hospital discharge education in ACS or its cost effectiveness. Translation of innovative, patient-centred, intervention strategies will contribute to the reduction of 30-day re-hospitalisation rates of patients discharged from the hospital after an ACS event [6,7,8].

In 2018, we conducted a systematic review of published randomised controlled trials and found only three studies, with one hundred and seventy-five participants evaluating discharge education strategies for ACS [9]. In these three studies, the impact of inpatient educational interventions was assessed in relation to attendance of cardiac rehabilitation programs, exercise rates and medication adherence. The intervention groups had increased attendance to cardiac rehabilitation compared to the control groups (47.3% vs. 21.1%, respectively). The intervention groups also showed increased exercise rates from baseline to follow-up compared to the control group (*p* = 0.049). No difference was reported between the intervention and control for medication adherence (*p* = 0.881). None of the three studies reported on re-admission, mortality or cost-effectiveness. This limited evidence for practice highlights the need for rigorous evaluations of novel ACS discharge patient education interventions [9].

Acknowledging the limited evidence for discharge education, the National Heart Foundation of Australia provides general recovery advice via its website and printed resources [10,11]. In this study we have used, as a conceptual framework, ‘The 6 Steps to Recovery” that the Heart Foundation advocate every nurse should discuss at the bedside before discharge [12]. The 6 Steps are: (1) Explain diagnosis and procedures; (2) Highlight risk factors relevant to the patient; (3) Emphasise the importance of cardiac rehabilitation; (4) Promote medication adherence; (5) Educate on the warning signs of heart attack and 6) Encourage follow-up with your doctor [12].

Other forms of post-discharge ACS education such as those presented at cardiac rehabilitation programs are often not accessed until several weeks after the event which may be past the point of buy-in for the patient. Additionally, the current uptake of cardiac rehabilitation (CR) and secondary prevention programs in Australia and around the world is between 20% and 46% [13]) so many ACS patients never receive this education. Previous research by our team has demonstrated a mismatch between access and attendance [14]. The majority of Australians have excellent geographic’ access to cardiac rehabilitation (CR) and secondary prevention programs after discharge, but this has not translated to attendance [15]. This research has indicated that addressing patients’ perceived need and cultural and gender differences might be key to improving attendance at CR [16]. Rather than fostering motivation and self-efficacy to attend existing programs, developing individualized self-care programs may be a more cost-effective solution for the uptake of CR [17].

### 1.1. Tracking Activity Levels and Acute Coronary Syndrome

Physical activity is a major component in a CR program and is integral to recovery from and ACS event [18]. This is primarily because physical activity has been shown to mitigate the progression of atherosclerosis which leads to ACS [19]. A substantial body of evidence shows that increased physical activity results in significant improvements in many ACS risk factors including hypertension, dyslipidaemia, obesity, insulin resistance and psychological health. However, these health benefits are only achieved when patients engage in the recommended total weekly amount of physical activity of at least moderate-intensity level for 150 minutes per week or more [19]. One of the main aims of CR is to encourage patients to achieve the recommended physical activity levels [10,18]. Cardiac rehabilitation also has medical, psychological, social and behavioral benefits and can increase functional capacity [19].

The use of wearable physical monitoring devices offers significant potential for researchers and clinicians working to promote or measure a patient’s activity levels. Activity tracking devices have the potential to effect physical activity behavior change, make a direct and real-time impact on self-management of physical activity and offer clinicians real world assessments of their patients’ daily activity patterns [20]. Wearable fitness tracking devices are ideal to use in the post-discharge ACS population to help track physical activity and to motivate sustained changes in moderate-intensity physical activity [21]. Current devices have become more user-friendly and can run for 5–7 days before a recharge is required [21].

Finally, global positioning systems (GPS) devices are now commonly used in society e.g., in high performance sport analysis, driving and even tracking movements of pets. Specialist software is required for the download and analysis of these data. The GPS data maps the distance and locations travelled and these can be matched to service locations. Unlike activity tracking devices, GPS devices do not differentiate travel by walking or vehicle nor educate or foster engagement with the tracking process.

### 1.2. Aim

The purpose of this research was to collect real-world data to determine if attendance to cardiac follow-up services was related to access (metro vs. rural and remote) in this important discharge period for ACS patients. The aim of this study was to also demonstrate the innovative use of GPS combined with geographic information systems (GIS) to objectively measure outcomes and explore if patients implemented recommended activity and utilisation of healthcare services (CR, pharmacy, general practice, pathology and emergency department) in the first 30 days and 90 days after discharge from hospital. The specific objectives of this study were to:

1. Determine the level of enthusiasm (satisfaction) of metropolitan and rural Aboriginal and Torres Strait Islander and non-Aboriginal and Torres Strait Islander cardiac patients to be involved in studies using tracking technology applications (apps).
2. Test the adherence of participants to using GPS and fitness tracking technology.
3. Compare the 30-day and 90-day post-discharge activity levels between those who lived in metropolitan areas with those who lived in rural areas.
4. Compare levels of attendance to cardiac healthcare services between patients who lived in areas with high access (metropolitan) and low access (rural).
5. Evaluate participant recall of discharge education and advice.

## 2. Materials and Methods

### 2.1. Study Design

To achieve our objectives, we used an observational design with quantitative and digital data collection (GPS and GIS) and qualitative patient-reported experiences (satisfaction survey) and a discharge education recall questionnaire.

### 2.2. Setting

Participants for the study were recruited as inpatients, between January 2018 and September 2018, from two metropolitan hospitals.

### 2.3. Participants and Recruitment

Participants were stratified to ensure representation from Aboriginal vs. Non-Aboriginal people and metropolitan and rural populations. Participants were recruited immediately prior to discharge. Aboriginal Liaison nurses assisted with the screening and recruitment of potential Aboriginal and Torres Strait Islander people.

### 2.4. Participant Inclusion Criteria

Eligibility criteria included: an index admission with a confirmed diagnosis of ACS; greater than 18 years of age, able to give consent; ability to speak, read and understand English; willingness to carry a phone for at least 30 days; and ability and willingness to receive a weekly phone call. Participants were ineligible to participate if they had cognitive impairment, were unable to participate in physical activity; had been admitted previously with ACS or were clinically unstable.

### 2.5. Intervention

Participants were educated on the use of the mobile phone activity tracking apps and requested to carry the phone for at least 30 days and up to 90 days. The phone was pre-installed with intervention software allowing for remote, virtual data collection. Participants were instructed to charge the phone overnight when they were asleep to avoid impacting on activity data collection. Participants were contacted weekly by the respective research nurses to assist with any technology issues.

### 2.6. Selection Criteria for Mobile Tracking Apps

This project was not about developing a mobile phone app. There were an overwhelming number of GPS and activity tracking mobile phone apps on the market at the time of this study. A Google search for GPS tracking apps revealed 7.25 million hits and 14.2 million hits for physical activity tracking apps. In this study there were three important criteria for selection of suitable commercially available mobile apps: (1) data could be collected from remote country locations and the data generated by the app had to be downloadable from a web portal, (2) battery usage, the apps needed to be able to run constantly on the phone for 12–16 h without the need for recharging and (3) low cost or free.

An unmodified ZTE 4GX smartphone mobile phone (ZTE Corporation and ZTE Australia), operating on a commercial satellite network was provided to all participants [22]. The device was loaded with our two preferred applications: Google Fit™– this free application collected daily step count (primary activity measure), calories consumed, distance travelled, geolocation, speed, inactive duration and active duration [23] and FollowMee™– this pay-per-user application collected geospatial data (primary measure), speed, altitude and distance travelled [24]. All app activity was hidden, and the device was password protected. Participants were permitted to keep their phone at the completion of their time in the study.

### 2.7. Variables/Data Sources/Measurement

Baseline data was collected from review of medical records and included age, postcode, gender, Aboriginal or Torres Strait Islander status, smoking status, household structure, height, weight, medical history, cardiac procedures, cardiac risk factors and comorbidities. Demographic data were collected in hard copy Case Report Forms (designed in Adobe LiveCycle Designer Enterprise Suite 3, (San Jose, CA, USA)) and emailed to the project manager on a weekly basis for entry into the database (Microsoft Excel 2016).

#### 2.7.1. Objective 1: Determine the Level of Enthusiasm for Being Involved in Studies Using Tracking Technology

“Enthusiasm” was determined by recruitment and adherence rates and a simple satisfaction survey developed for this study to measure patient-reported experiences measures (PREMS).

#### 2.7.2. Objective 2: Test the Adherence of Participants to Using GPS and Fitness Tracking Technology

Downloaded activity and GPS data was used to measure adherence by evaluating missing data, gaps in recording and periods of non-activity.

#### 2.7.3. Objective 3: Compare the 30-Day and 90-Day Post-Discharge Activity Levels between Those Who Live in Metropolitan Areas with Those Who Live in Rural Areas

To compare the 30 to 90-days post-discharge activity levels of metropolitan participants to rural, the Google Fit app was used to collect daily step counts. Activity data were emailed daily to the research team from the respective applications in comma separated values (CSV) format. The daily step data obtained from the Google Fit application were aggregated and mean and median steps per person per day were calculated for the periods of 1–30, 31–60 and 61–90 days (the latter period data were adjusted to reflect those who exited the study prior to 90 days). A total of 151,859 rows of raw data were generated in CSV format from this study.

#### 2.7.4. Objective 4: Compare Levels of Access to Cardiac Healthcare Services between Patients Who Lived in Areas with High (Metropolitan) and Low (Rural)

Participant home locations were stratified using the accessibility and remoteness index of Australia (ARIA) to determine rurality. Participants were asked to give the address of their local services (CR, pharmacy, general practice, pathology and emergency department) at base-line and these were mapped using VERITAS^TM^ software (Veritas Technologies LLC, Santa Clara, CA, USA). The FollowMee™ application collected geospatial data (primary measure). Descriptive statistics were generated for the accuracy of the data point and stratified by type (GPS or Network) and a rural or metropolitan classification of the participant. A summary of data points, categorised by time, were produced to give an indication of device usage over the project, as was a summary of data availability based upon accuracy of data point. A total of 153,000 lines of raw data cleaned to 45,577 were generated in CSV format during the study period.

The GPS tracking app was required to have a two-minute ping option (track interval), while the activity tracking app needed to include a step counter and measure of distance travelled. The FollowMee™ GPS tracking application featuring a two-minute ping combined with the ability to automatically suspend tracking when the participant was inactive and reactivate upon movement offered both battery efficiency and a high level of location accuracy. Similarly, the GoogleFit™ application offered high accuracy with the required activity tracking features while conserving battery power. The cost of subscriptions for both applications needed to access all the required features, were moderate. The use of these applications facilitated the use of a single device by the participants without the need for manual data download or return of the device streamlined the data collection process significantly. The GPS data mapped the distance and locations travelled by the participants and were matched to service locations. The street addresses were geocoded and based on the accuracy of the data points within each of seven arbitrary buffers zone (See Table 1) a 50 m buffer was applied. Patients whose FollowMee™ track data that entered the 50 m buffer zone were recorded as attended for that health service.

#### 2.7.5. Objective 5: Evaluate Participant Recall of Discharge Education and Advice

Immediately prior to discharge patients were interviewed by the research nurse who administered (face-to-face) a questionnaire summarising “The 6-Steps to Recovery” [12]. The questions included asking the participants to explain their diagnosis (in their own words) and any other conditions or risk factors they thought may affect their heart. The questionnaire asked participants to recall any procedures performed during their admission as well as any written information received regarding exercise, diet, medication, driving and follow-up appointments. Participants were asked if they had an appointment with their general practitioner, whether a supply of medications been given, had a script for medications been given, whether new medications had been prescribed and if they had received a referral to a cardiac rehabilitation program.

### 2.8. Study Size

Given this was intended to act as a feasibility study to inform a larger project, the study size was determined primarily by the funding timeframe (12 months). This was broken down into device and application testing which ran concurrently with ethical and governance submission (3 months), recruitment period (4 months) follow-up (up to 90 days post-enrolment) and analysis and manuscript preparation (2 months).

### 2.9. Follow-Up

If participants adhered to the protocol their data was uploaded from the device. Follow-up was attempted once each week on those who were uncontactable at their allocated appointment time. Those who failed four consecutive follow-up calls were considered lost to follow up.

### 2.10. Analysis

Data management were undertaken in Microsoft Excel 2016. All spatial analyses were carried out in ArcGIS Desktop 10.2.2 (Environmental Systems Research Institute, (ESRI) Redlands, CA, USA) and statistical analyses were undertaken in IBM SPSS for Windows, Version 22. Summary statistics were used to describe data from on the characteristics of the participants of different groups. Cronbach’s alpha was used to quantify interrater reliability between self-report and medical record data. Subgroups were stratified as (1) rural/remote or metropolitan and (2) self-report or medical record. Missing spatial data was unable to be accounted as it relied upon adherence to the study protocol by participants. A summary of data points stratified by time provides is indicative of device adherence. Data accuracy in meters was collected, as was the source (GPS or Network). Stratification of available data were used to guide the final dataset used for analyses (data points with an accuracy of ≤20 m were retained for the spatial analysis). Statistical tests were not used to compare groups due to low overall participant numbers.

### 2.11. Ethics

Health Research Ethics Committee approval was given from both recruitment sites (HREC/17/RAH/58 and R20170222) with additional Aboriginal Health Research Ethics Committee approval AHREC Protocol #: 04-17-714. Activity reported in this study was strictly confined to physical activity and attendance at cardiac related health services during the 30 to 90-day post-discharge period.

## 3. Results

### 3.1. Recruitment

**Figure 1:**
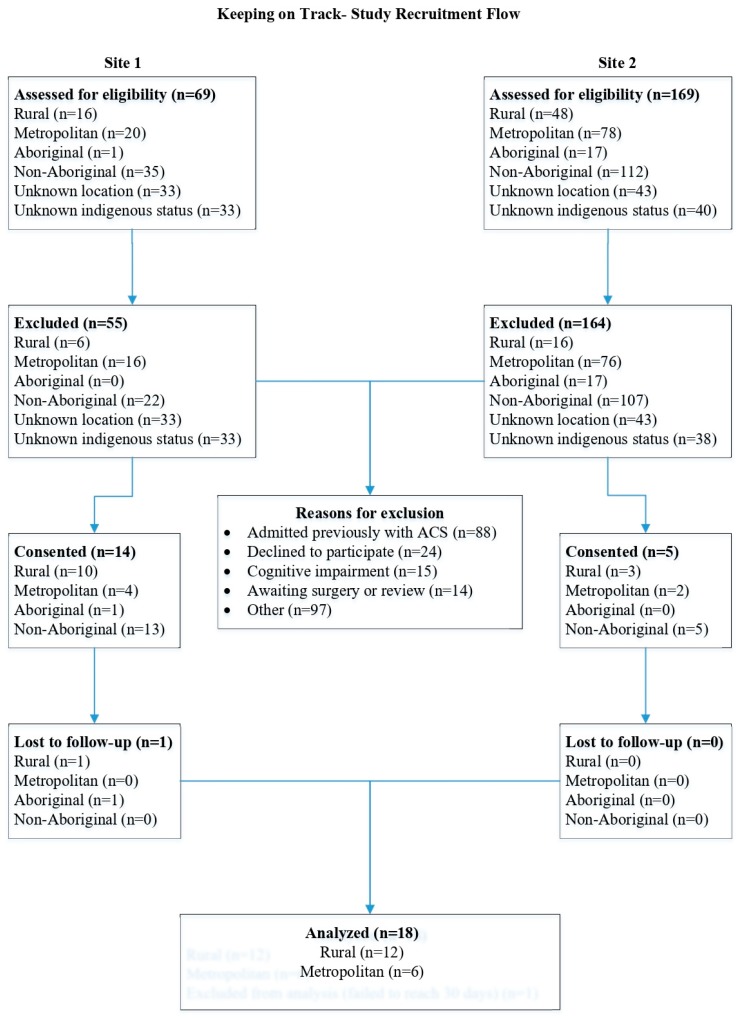
Study recruitment flow chart.

During the study period, 238 participants were screened including 18 Aboriginal and Torres Strait Islander people. Reasons for exclusion included: admitted previously with ACS (37% of the total and 16 of the potential Aboriginal people screened); people who declined to participate (10%); cognitive impairment (6%); awaiting surgery or review (5%) and other included being enrolled in another study (41%). At completion of the recruitment period, 19 participants had consented including two Aboriginal and Torres Strait Islander people. One of the Aboriginal participants was later lost to follow-up which left only one Aboriginal person participating, consequently this person’s data has not been reported separately (Figure 1). Although our intention was also to have 50:50 metropolitan to rural and remote, the final cohort included six (33.3%) metropolitan and 12 (66.6%) rural and remote participants.

### 3.2. Demographic and Clinical Characteristics of Participants

The mean age of the participants was 55.8 years. The majority were male (83%) (metro 27.8% vs. rural 55.6%) and lived with a partner or carer (88.9%) (Table 2). The participants had a mean of four CVD risk factors per person (range 2–8) including being overweight or obese (72%) current or ex-smokers (72%), hypertensive, (38.9%) and hypercholesterolemia (61%). Sixty-one percent were diagnosed as ST-elevation myocardial infarction (Table 2).

### 3.3. Enthusiasm and Satisfaction with Being Involved in Studies Using Tracking Technology Applications

At the recruitment phase enthusiasm was high with only 10% of those approached declining to participate. This participant group reported highly satisfactory experiences with participating in the study. Satisfaction was measured on completion of follow-up at 30 days (87%) and 90 days (90%).

### 3.4. Adherence of Participants to Using Global Positioning Systemsand Fitness Tracking Technology

Downloaded activity and GPS data was used to measure adherence by evaluating missing data, gaps in recording and periods of non-activity. All except one participant maintained (charged) the phones and ensured data transmission. Consequently, at completion of the study, 18 (94%) participants had provided 30 days of tracking data and 15 (83%) participants had provided 90 days of tracking data.

### 3.5. Compare the 30-Day and 90-Day Post-Discharge Activity Levels between Those Who Lived in Metropolitan Areas with Those Who Live in Rural Areas

**Figure 2:**
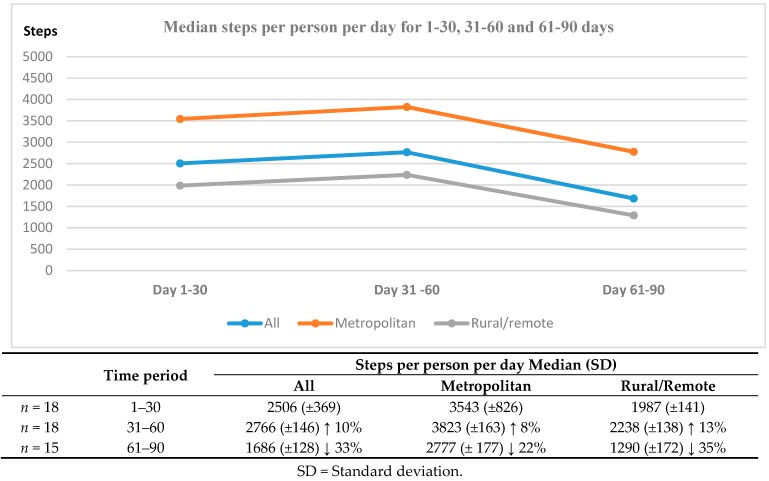
Participants objectively measured physical activity.

Total physical activity recorded for all participants in the was a median of 2506 (standard deviation (SD) ± 369) steps per day in the first 30 days, slightly higher for metro participants (median 3543 (SD ± 826) steps) and slightly less for rural participants (median 1987 (SD ± 141) steps). Two thousand five hundred steps per day was the equivalent of 20 min of walking or approximately 1.5 km (Figure 2).

**Figure 3:**
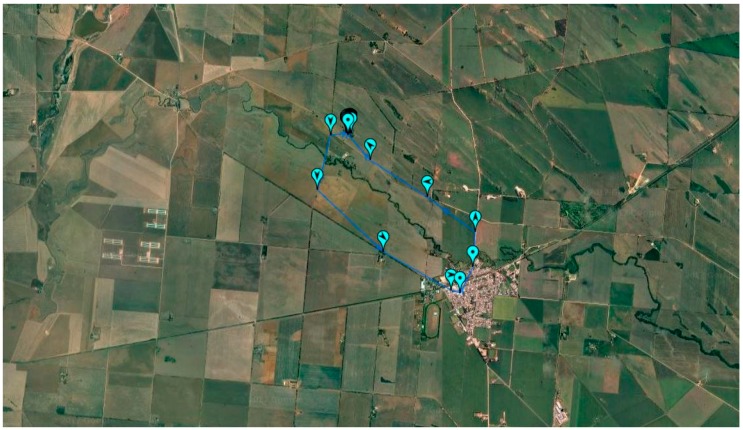
Global positioning systemmap of participant walk in the country.

There was an overall 10% increase in physical activity in the next 30 days, but this was not sustained. By 90-days post-discharge the total physical activity as measured by steps-per-day had significantly decreased (*p* < 0.05) by 33% to 1686 (±128) steps per day (metro ↓ 22% 2777 (±177) steps vs. rural and remote ↓ 35% 1290 (±172) steps). There was one rural outlier who completed 10,000 steps per day from seven days post-discharge. One of these daily walks demonstrating the maps produced by the GoogleFit™ app is presented in Figure 3.

### 3.6. Compare Levels of Access to Cardiac Healthcare Services between Patients Who Lived in Areas with High (Metropolitan) and Low (Rural)

The FollowMee™ application collected geospatial data. The mean accuracy of GPS captured data points was at 16.2 m (95% CI, 15.92–16.45 m, median 7 m) compared to the mean accuracy of satellite-captured data which was within 1832.7 m (95% CI, 1774—1891 m, median 2059 m). For rural participants, the mean accuracy of GPS data points was within 98 m (95% CI, 92.6–103.5 m, median = 9 m, with 96% of the GPS data captured) and for metropolitan participants the mean accuracy of GPS data capture was 33.5 m (95% CI, 31.2–35.9, median 6 m, 98.1% GPS-captured). Based upon the summary of data availability stratified by the accuracy of the data point, only data points with an accuracy of ≤20 m were retained for the spatial analysis (81.3% of all data available). This was a balance between enough rigor and utilising the data available.

Access to cardiac related health services in the first 30 to 90 days post-discharge is presented in Table 3. In the first 30 days 67% of participants had visited their GP (metro 25% vs. rural 37.5%), 72% had visited a pharmacy, 28% had present to an emergency department and 16.7% had seen their cardiologists. By 90 days 25% had attended cardiac rehabilitation (metro 16.7% vs. rural 8.3%) and there was no GPS recorded attendance to see a cardiologist during the 30–90-day period. 

### 3.7. Evaluate Participant Recall of Discharge Education and Advice

To evaluate participant, recall of discharge education and advice the Heart Foundation 6 Steps to Recovery discharge education framework was used. The 6 Steps are: (1) Explain diagnosis and procedures; (2) Highlight risk factors relevant to the patient; (3) Emphasise the importance of cardiac rehabilitation; (4) Promote medication adherence; (5) Educate on the warning signs of heart attack and (6) Encourage follow-up with your doctor [12] (Table 4 and Table 5).

(1) Explain diagnosis and procedures: When questioned immediately prior to discharge 100% of the participants could recall their diagnosis, 88% of participants who had a stent indicated they had this procedure and a mean of 24% participants could recall other procedures conducted during the admission.

(2) Highlight risk factors relevant to the patient: Ninety percent of participants knew they had a family history of CVD, 100% knew they had diabetes, 60% hypertension and 80% hypercholesterolaemia. Sixty percent recalled being given advice about exercise, diet and follow-up appointments and 70% recalled being given advice regarding medications. Ninety percent of participants recalled the advice they were given about driving. With respect to understanding which risk factors could affect their heart, one participant (5.6%) had knowledge regrading diabetes, hypertension and hypercholesterolemia, three (16.7%) understood about the impact of body weight, four (22.2%) smoking and two indicated they thought stress was a risk factor.

(3) Emphasise the importance of cardiac rehabilitation: According to the GPS tracking data, at 30-days post-discharge, twp (16.7%) participants (one metro vs. one rural) had attended the location where a cardiac rehabilitation service was located. In the period up to-90 days a total of three (25%) participants (two metro vs. one rural) had attended a cardiac rehabilitation service (Table 5).

(4) Promote medication adherence: At the 0–30day timepoint 13 (72.2%) had been tracked to their local pharmacist location at 31–90 days 11 (61.1%) participants had attended.

(5) Educate on the warning signs of heart attack: Eighty eight percent of the participants recalled receiving warning signs information contained in the discharge information booklet.

(6) Encourage follow-up with your doctor: Table 5 demonstrates that in the period from 0 to 30 days post-discharge, 10 (62.5%) had been GPS located to the address of their nominated general practice and a further seven (43.8%) in the period from 31 to 90days discharge.

## 4. Discussion

The purpose of this research was to collect real-world data and determine if access to services had an impact on utilisation in this important discharge period for ACS patients. The aim of this study was also to demonstrate the innovative use of GPS combined with GIS to objectively measure outcomes and explore if patients implemented recommended activity and utilisation of healthcare services (CR, pharmacy, general practice, pathology and emergency department) in the first 30 days and 90 days after discharge from hospital.

Our recruitment plan was to recruit DiNovo ACS patients at discharge including fifty percent Aboriginal and Torres Strait Islander people and non-indigenous people equally from metro and rural and remote areas. Review of our recruitment data indicated that during the recruitment period sixteen of the eighteen potential Aboriginal people screened had had a previous ACS event and were therefore not eligible to participate.

The demographic and clinical characteristics of participants mean age of the participants was 55.8 years, the majority were male, 60% STEMI mean of four risk factors per person (range 2–8) were consistent with other studies reporting the characteristics of Australia ACS patients [25,26].

At the recruitment phase enthusiasm was related high with only 10% of those approached declining to participate. This group, which was mostly male, reported high levels of satisfaction with participating in the study. Our conclusion to the high levels of enthusiasm was the participation incentive of a free phone. However, this was moderated by adherence to the study protocol. At completion of the study, 18 (95%) of participants had provided 30 days of tracking data and 15 (83%) of participants provided 90 days of tracking data. This rate was high compared to our recent systematic review [27] that examined adherence to physical activity tracking apps. In this study, we reported that, within the ten eligible studies reviewed, there was a mean adherence rate to using the device of 59.1% (range 39.6% to 85.7%). Consistent with our study this review also reported that current studies of physical activity using apps lacked equal representation by gender (28.6% female) and age (range 42 to 82 years). Our systematic review indicated that current research on activity monitoring devices may be overstated due to the variability in adherence. Results show physical activity tracking in women and young adults has been understudied [27].

Satisfaction with the experience of being involved with this study was high (30 days (87%) and 90 days (90%). Patient-reported measures (PREMS) collect information about the experience in a research study, and the outcomes of research involvement, as described by patients. Interest is now growing in strengthening and coordinating efforts to collect patient-reported information to benefit patients participating in health research [28].

At discharge patients with a diagnosis of ACS are advised to aim to build up to doing at least thirty minutes of moderate-intensity physical activity on most, if not all, days of the week. This can be achieved in three lots of ten minutes each if it’s easier for example ten minutes each of walking, gardening and light housework [10] in this study, participants were always asked to carry the phone on their person during the day, data shows adherence to this was high (87%). Therefore, our results show physical activity goals were not achieved by many of our participants. Very few studies have looked at physical activity without active intervention. One study from Germany has confirmed in a cohort of 29,000 patients, only 9% of post-myocardial infarction and 5.5% of stroke patients achieved their secondary prevention physical activity goals. Our knowledge questionnaire also demonstrated that at discharge only sixty percent of patricians recalled information given about exercise.

Access to cardiac related health services in the first 30 to 90 days post-discharge could be described as moderate to low. Attendance at a cardiac rehabilitation program which was sixteen percent compared to national average of twenty to forty percent. Additionally, only sixty percent of participants could recall being told about the importance of adhering to medication and keeping their appointments with doctors.

In Australia, approximately 96% of the population live within one-hour drive time of the four basic services to support cardiac recovery and secondary prevention, including 96% of older Australians and 75% of the indigenous population. A study measuring access to CVD secondary prevention health services, demonstrated that most Australians have excellent geographic access to services to support cardiac recovery and secondary prevention. Therefore, it appears that it is not the distance to services that affect attendance. Our geographic lens has identified that more research on socioeconomic, sociological or psychological aspects to attendance is needed [14].

Participant recall of the discharge information was variable. All participants and all participants with diabetes knew their diagnosis. The CVD risk factors were less understood and discharge advice seemed to reflect only high-level recall of the issues immediately and directly affecting participant lifestyles, for example ninety percent (range 88–94%) recalled being told about driving limitations but only sixty percent could remember being told about diet, exercise and follow-up appointments. These results reflect the outcomes reported previously where only one-quarter of all patients admitted with ACS received optimal secondary prevention (pharmacotherapy, lifestyle advice, and referral to rehabilitation) [26]. The SNAPSHOT ACS study in 2013 [25] provided unique insights into the provision of lifestyle modification advice (or lack thereof) to patients admitted to hospital with an ACS. These findings highlight the persistence of the evidence treatment gaps in a contemporary cohort of ACS patients. Standardising inpatient care in-line with guidelines was recommended [26].

## 5. Limitations and Lessons Learned

The main limitations of this study were sample size and follow-up time frames which limits the generalizability of the results. However, this was a feasibility study to inform a larger project, the study size was determined primarily by the funding timeframe of 12 months.

Although it was the intention of this study to recruit fifty percent Aboriginal people due to previous ACS admissions the majority who presented during our limited recruitment period were ineligible. The lesson learned from this was that a longer recruitment period would be required for future studies.

Managing Big Data from the tracking apps was a challenge with thousands of data points delivered daily and a large amount of research funding consumed to manage and prepare these data for analysis. A lesson learned from this experience would be that for future similar studies the budget must include funding for a data manager whose role is to solely tend to Big Data management. Logistical issues also occurred in managing an individual email account for each phone/participant from the GoogleFit^TM^ app.

Some mobile phones exhibited poor battery life and some participants forgot to charge the phones nightly; this was remedied by the addition of a nightly text reminder service.

Weekly follow-up calls were also a workload burden even for this small group. Since the apps were installed on mobile phones, we would recommend text messages communication for future studies.

Due to the co-location of many community health services data may have overestimating access to cardiac-related healthcare services.

Most data points were captured by GPS. Rural participants had a higher proportion of network-capture than metropolitan participants due to line of sight from their location to the mobile phone tower.

GPS tracking devices did not differentiate travel by vehicle or educate or foster engagement with the tracking process.

Both apps (FollowMee^TM^ and GoogleFit^TM^) were off the shelf and offered limited customizability; furthermore, the FollowMee^TM^ app was prone to crashing several times a week for some users.

Finally, the naming of this study was a lesson learned. The original title given to the study was “The Tracker Study”. Early in the recruitment phase we were informed that a “Tracker” is a euphemism for ankle bracelet home detention devices. After consultation with the Wardliparringga Aboriginal Research Unit the study title was modified.

## 6. Conclusion

This study was an example of the application of objective data from the real world to help understand post-ACS discharge patient activity. Rates of access to services in the first 30 days continue to be of concern. The outcomes of this exploratory work will provide an understanding of the impact of discharge education after ACS hospitalisation. The preliminary data collected from this study will be used to develop innovative, patient-centred, interventions that can improve outcomes by improving adherence to secondary prevention strategies and reduce 30-day re-hospitalisations and mortality.

## Figures and Tables

**Table 1:** Buffer Zone accuracy.

Buffer Zone (meter)	Data Points (*n*)	Data Accuracy (%)
5	18,096	43.1
10	27,643	65.9
20	34,097	81.3
30	35,621	84.9
40	36,555	87.2
50	37,468	89.3
100	39,157	93.4

**Table 2:** Demographics, clinical characteristics and risk factors of participants.

Demographics/Risk Factors	Total *n* = 18 *n* (%)	Metropolitan *n* = 6 *n* (%)	Rural/Remote *n* = 12 *n* (%)
Mean age (years)	55.8	55.0	56.3
≤44 years	4 (22.2)	2 (11.1)	2 (11.1)
45 to 65 years	8 (44.4)	2 (11.1)	6 (33.3)
>65 years	6 (33.3)	2 (11.1)	4 (22.2)
Gender (male)	15 (83.3)	5 (27.8)	10 (55.6)
Lives with partner, relative or carer	16 (88.9)	6 (33.3)	10 (55.6)
* Mean body mass index (BMI) kg/m^2^	29.7	28.0	30.6
* Overweight or obese > BMI 25 kg/m^2^	13 (72.2)	4 (22.2)	9 (50.0)
Current or previous smoker	13 (72.2)	4 (22.2)	9 (50.0)
Type II Diabetes	3 (16.7)	-	3 (16.7)
Hypertension	7 (38.9)	2 (11.1)	5 (27.8)
Hypercholesterolemia	11 (61.1)	5 (27.8)	6 (33.3)
Family history of cardiovascular disease	10 (55.6)	2 (11.1)	8 (44.4)
Total number of risk factors			
At least one risk factor Median Range	18 (100.0)	6 (33.3)	12 (66.7)
	4.0	4.0	4.5
	2–8	2–6	2–8
Cardiac Diagnosis			
ST-elevation myocardial infarction	11 (61.1)	4 (22.2)	7 (38.9)
Non-ST-elevation myocardial infarction	5 (27.8)	1 (5.3)	4 (22.2)
Anterior myocardial infarction	1 (5.3)	1 (5.3)	-
Infero-posterior myocardial infarction	1 (5.3)	-	1 (5.3)
Procedures performed in hospital			
Angiogram	17 (94.4)	6 (33.3)	11 (61.1)
Angioplasty	2 (11.1)	2 (11.1)	-
Echocardiogram	10 (55.6)	3 (16.7)	7 (38.9)
Magnetic resonance imaging	1 (5.3)	-	1 (5.3)
Percutaneous coronary stent insertion	16 (88.9)	6 (33.3)	10 (55.6)
Thrombolytic therapy	3 (16.7)	-	3 (16.7)

* World Health Organization (WHO) Adapted from WHO, 1995, WHO, 2000 and WHO 2004. m^2^ = square meters.

**Table 3:** Participant access to services at 30 and 90 days.

Health Service Type	Location Provided *n* (%)	Number of Participants That Visited Location 1,2 0–30 Days *n* (%)			Number of Participants That Visited Location 1,2 31–90 Days *n* (%)		
		All	Metropolitan	Rural/Remote	All	Metropolitan	Rural/Remote
Gen Practitioner	16 (88.9)	10 (62.5)	4 (25.0)	6 (37.5)	7 (43.8)	2 (12.5)	5 (31.3)
Card rehab	12 (66.7)	2 (16.7)	1 (8.3)	1 (8.3)	3 (25.0)	2 (16.7)	1 (8.3)
Cardiologist	18 (100)	3 (16.7)	0 (0.0)	3 (16.7)	0 (0.0)	0 (0.0)	0 (0.0)
Pharmacy	18 (100)	13 (72.2)	5 (27.8)	8 (44.4)	11 (61.1)	3 (16.7)	8 (44.4)
Community Health Service	3 (16.7)	3 (100)	1 (33.3)	2 (66.7)	2 (66.7)	0 (0.0)	2 (66.7)
ED	18 (100)	5 (27.8)	1 (5.6)	4 (22.2)	3 (16.7)	0 (0.0)	3 (16.7)
Pathology	18 (100)	11 (61.1)	5 (27.8)	6 (33.3)	3 (16.7)	2 (11.1)	1 (5.6)

The participant’s attendance was recorded if they entered a zone within a 50 m radius at the provided locations. Some health services were co-located. Gen Practitioner = general practitioner; Card rehab = cardiac rehabilitation; ED = emergency department.

**Table 4:** Baseline knowledge of conditions & procedures & understanding of discharge advice.

Item	Stated by Participant (*n* = 18) *n* (%)	Recorded in Medical Records *n* (%)	Interrater Reliability Rate (% match)
Discharge diagnosis	18	18	100%
Knowledge of medical history			
Type II Diabetes	3 (16.7)	3 (16.7)	100%
Hypertension	4 (22.2)	7 (38.9)	60%
Hypercholesterolaemia	9 (50.0)	11 (61.1)	80%
Family History of CVD	9 (50.0)	10 (55.6)	90%
Total score (mean)	6.3 (35.0)	7.8 (43.3)	80%
Knowledge of procedures performed during current admission			
Angiogram	6 (33.3)	17 (94.4)	40%
Angioplasty	0 (0.0)	2 (11.1)	0%
Echocardiogram	4 (22.2)	10 (55.6)	40%
MRI	0 (0.0)	1 (5.6)	0%
Percutaneous coronary stent insertion	16 (88.9)	16 (88.9)	100%
Thrombolytic therapy	0 (0.0)	3 (16.7)	0%
Total score (mean)	4.3 (24.1)	8.2 (45.6)	30%
Knowledge of educational materials/and information received			
A copy of Discharge Booklet received	15 (83.3)	16 (88.9)	90%
Any other written information	7 (38.9)	12 (66.7)	60%
Knowledge of advice given about what to do when they go home			
Exercise	9 (50.0)	16 (88.9)	60%
Diet	8 (44.4)	13 (72.2)	60%
Medication	12 (66.7)	18 (100)	70%
Driving	16 (88.9)	17 (94.4)	90%
Appointments	11 (61.1)	18 (100)	60%
Total score (mean)	11.2 (62.2)	16.4 (91.1)	70%
Knowledge conditions that can affect the heart			
Diabetes	1 (5.6)		
Hypertension	1 (5.6)		
Hypercholesterolemia	1 (5.6)		
Weight	3 (16.7)		
Smoking	4 (22.2)		
Stress	2 (11.1)		
Total score (mean)	2 (11.1)		
Participants recall regarding referral to cardiac rehabilitation	13 (72.2)		

MRI: magnetic resonance imaging.

**Table 5:** Overall scorecards for adherence to discharge advice (6 steps).

No.	Item	Source	Question/Activity	*n* (%) Achieved or Recalled
1.	Know your diagnosis and the cardiac procedures you have had	Knowledge quiz (Table 3)	Diagnosis	18 (100)
			Procedures	4.3 (24.1)
2.	Understand your risk factors	Knowledge quiz (Table 3)	Medical history	6 (35.0)
			Conditions that can affect the heart	2 (11.1)
			Discharge advice	11.2 (62.2)
3.	Go to cardiac rehabilitation	Global Positioning System (GPS) data	Attended CR location at 0–30 days	2 (16.7)
			Attended CR location at 31–90 days	3 (25.0)
4.	Take your medications	GPS data: visits to nominated pharmacist location	Tracked to pharmacist location at 0–30 days	13 (72.2)
			Tracked to pharmacist location at 31–90 days	11 (61.1)
5.	Know your warning signs of heart attack and respond appropriately	“My Heart My Life” Heart Foundation booklet	Was a copy of “My Heart My Life” received	16 (88.9)
6.	See your doctor (General Practitioner) regularly	GPS data: Visits to nominated GP location	No. of visits to GP 0–30 days	10 (62.5)
			No. of visits to GP 31–90 days	7 (43.8)
